# Surface Triggered Self-Assembly of Fmoc-Tripeptide as an Antibacterial Coating

**DOI:** 10.3389/fbioe.2020.00938

**Published:** 2020-08-07

**Authors:** Miryam Criado-Gonzalez, Muhammad Haseeb Iqbal, Alain Carvalho, Marc Schmutz, Loïc Jierry, Pierre Schaaf, Fouzia Boulmedais

**Affiliations:** ^1^Université de Strasbourg, CNRS, Institut Charles Sadron UPR 22, Strasbourg, France; ^2^Institut National de la Santé et de la Recherche Médicale, UMR-S 1121, “Biomatériaux et Bioingénierie”, Strasbourg, France; ^3^Faculté de Chirurgie Dentaire, Fédération de Médecine Translationnelle de Strasbourg and Fédération des Matériaux et Nanoscience d’Alsace, Université de Strasbourg, Strasbourg, France

**Keywords:** peptides, hydrogels, antimicrobial, enzyme-assisted self-assembly, nanoparticles

## Abstract

In western countries, one patient on twenty will develop a nosocomial infection during his hospitalization at health care facilities. Classical antibiotics being less and less effective, this phenomenon is expanding year after year. Prevention of bacteria colonization of implantable medical devices constitutes a major medical and financial issue. In this study, we developed an antibacterial coating based on self-assembled Fmoc-tripeptide. Fmoc-FFpY peptides (F: phenylalanine; Y: tyrosine; p: PO_4_^2–^) are dephosphorylated enzymatically into Fmoc-FFY by action of alkaline phosphatase functionalized silica nanoparticles (NPs@AP), previously deposited on a surface. Fmoc-FFY peptides then self-assemble through π–π stacking interactions, hydrogen bonds and hydrophobic interactions adopting β-sheets secondary structures. The obtained hydrogel coatings show fibrillary structures observed by cryo-scanning electron microscopy with a thickness of few micrometers. At low concentration (≤0.5 mg.mL^–1^), self-assembled Fmoc-FFY has a superior antibacterial activity than Fmoc-FFpY peptide in solution. After 24 h of incubation, Fmoc-FFY hydrogel coatings fully inhibit the development of Gram-positive *Staphylococcus aureus (S. aureus)*. The antibacterial effect is maintained on an *in vitro* model of repetitive infection in the case of *S. aureus*. This coating could serve in infections were Gram positive bacteria are prevalent, e.g., intravascular catheter infections. This work gives new insights toward the design of an alternative antimicrobial coating.

## Introduction

Biomedical implants, i.e., prosthetics, catheters or intraocular lenses, are indispensable in medicine and gain increasing attention over the years (Lombardi et al., 2019). However, the direct contact of their surfaces with biological fluids becomes susceptible to bacterial colonization and biofilm formation leading to a major medical and financial issue (McCloskey et al., 2014). Implant-associated infections increase the probability of implant failure and may result, if untreated, in chronic microbial infection, inflammation, tissue necrosis, and even morbidity (McCloskey et al., 2014). In western countries one patient on twenty will develop a nosocomial infection during his hospitalization at health care facilities (Cassini et al., 2016). These infections are mostly due to *Staphylococcus epidermidis*, in the case of intravascular catheter-associated infections (Rupp and Archer, 1994), and *Staphylococcus aureus* (*S. aureus*) in the case of metallic implants (Barth et al., 1989). Biofilm formation contributes to the resistance to antibiotic treatments, it protects the bacterial colonies from host defense systems and bactericidal agents (Kaplan, 2011). This leads to the chemotherapeutic failure which often results in an increase of the resistance mechanism adopted by many bacterial strains, particularly those involving *S. aureus* (Darouiche, 2004). In this scenario, the development of antimicrobial coatings to protect against such infections has become a major field of scientific and technological research (Séon et al., 2015). As alternative to antibiotics, antimicrobial peptides are promising candidates to overcome pathogen resistance and for clinical exploitation (Lombardi et al., 2019). Self-assembly of peptides, with a sequenced-defined chemical structure, can give hydrogels with a desired functionality which becomes increasingly attractive in the development of therapeutics materials (Makam and Gazit, 2018; Criado-Gonzalez et al., 2020a).

In the last decade, low molecular weight hydrogelators (LMWH) fabricated from natural biomolecules such as amino acids got great interest due to their ease and powerful bottom-up fabrication. They self-assemble into supramolecular hydrogels by non-covalent interactions, i.e., electrostatic, van der Waals forces, hydrogen bonding and π-π stacking (McCloskey et al., 2014), in response to external stimuli, such as temperature change (Collier et al., 2001), pH switch (Aggeli et al., 2003; Chen et al., 2011), solvent change (Mahler et al., 2006), electrostatic interactions (Criado-Gonzalez et al., 2020b), chemical (Bowerman and Nilsson, 2010; Zhao et al., 2011) or enzymatic reactions (Yang et al., 2004; Yang and Xu, 2006; Criado-Gonzalez et al., 2019b). Ultrashort peptides, up to 7 amino acids in length, are an emerging class of LMWH of increasing interest due to their versatility in molecular design, ease synthesis, and costs reduction. Few studies have been carried out on the development of self-assembling materials with antibacterial properties using short peptides. Based on the pioneering work of Moir and coll. (Soscia et al., 2010; Kumar et al., 2016) which demonstrated the antimicrobial activity of an amyloid-β peptide, Schnaider et al. (2017) proved the antibacterial activity of a self-assembled dipeptide, diphenylalanine (FF), against *Escherichia Coli* (*E. coli*). In this work, FF moiety was identified as the central recognition module of amyloid, as well as the fundamental self-assembly motif proving the efficiency of this unit for the development of self-assembling antimicrobial hydrogels. Fluorenylmethyloxycarbonyl-FF (Fmoc-FF) derived peptides were shown to possess excellent activity against the most antibiotic resistant biofilm phenotype of both Gram-positive and Gram-negative bacteria (McCloskey et al., 2017). Fmoc-FF self-assemblies were employed as host hydrogels to incorporate silver nanoparticles to improve the antimicrobial response (Paladini et al., 2013). The backbone of this dipeptide was modified incorporating an urea moiety to inhibit the *E. coli* bacterial adhesion (Basavalingappa et al., 2019).

Poorly soluble in water, Fmoc-FF peptide self-assembly is induced by dissolution in DMSO followed by a dilution step in water (Basavalingappa et al., 2019), by change of pH (Paladini et al., 2013), or by heating the solution, up to 90°C for solubilization, followed by a cooling step (Kumar et al., 2016). To overcome these tedious processes, the enzyme assisted self-assembly (EASA) of peptides was introduced by Xu and co-workers (Yang et al., 2004; Yang and Xu, 2006). Soluble at room temperature in aqueous solution, phosphorylated peptides were transformed into gelators by alkaline phosphatase (AP) catalyzing the removal of the phosphate groups. Later, we introduced the use of non-self-assembling Fmoc-FFpY peptide (Y: tyrosine; p: PO_4_^2–^), which is transformed into the hydrogelator Fmoc-FFY by AP. The enzymatic assisted self-assembly of Fmoc-FFpY was localized on a planar substrate (Vigier-Carrière et al., 2017) or on silica nanoparticles (Criado-Gonzalez et al., 2019a) thanks to the functionalization of their surfaces by AP. In contrast to the pH triggered self-assembly (Jayawarna et al., 2006; Raeburn et al., 2012), the EASA allows to use a more water-soluble peptide as a precursor and to obtain, by simple contact with the peptide solution, a fast and localized self-assembly on the surface of a substrate, previously functionalized by the enzyme.

**SCHEME 1:**
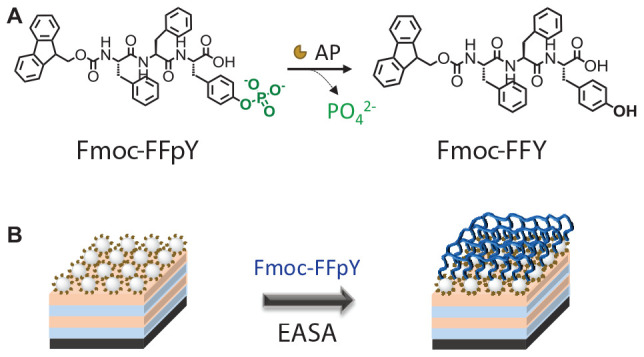
Schematic representation of Fmoc-FFY hydrogel coating: **(A)** Enzyme-assisted self-assembly (EASA) of Fmoc-FFY by Fmoc-FFpY dephosphorylation **(B)** obtained on the surface of NPs@AP coating deposited on polyelectrolyte multilayer film.

Integrating self-assembling peptides with antimicrobial property on the surface of a biomaterial is an innovative design for the development of antibacterial materials. Herein, we report an antibacterial coating based on Fmoc-FFY hydrogel self-assembled by enzymatic dephosphorylation. To this aim, AP functionalized silica nanoparticles (NPs@AP) were first deposited on the surface of a material using the layer-by-layer method and then put in contact with Fmoc-FFpY solution to obtain the Fmoc-FFY hydrogel coating (Scheme 1). NPs@AP allowed to immobilize a higher quantity of enzyme on the surface than AP monolayer. The antimicrobial property was tested against *S. aureus*, Gram-positive strain which is one of the most virulent bacteria leading to high rates of device-related systemic infections and mortality and *E. coli*, a Gram-negative strain mainly found on the surface of urinary catheters.

## Materials and Methods

### Materials

Poly(ethylene imine) (PEI, Mw = 250 000 g.mol^–1^), Poly(allylamine hydrochloride) (PAH, M_*w*_ = 58 000 g.mol^–1^), Alkaline Phosphatase (AP, 10 DEA units.mg protein^–1^) from bovine intestinal mucosa, Poly(sodium 4-styrenesulfonate) (PSS, M_*w*_ = 70 000 g.mol^–1^), Tetraethyl orthosilicate (TEOS), (3-glycidyloxypropyl) trimethoxysilane (GPMS) were from Sigma Aldrich. *p*-Nitrophenyl phosphate (PNP) was from ThermoFisher Scientific. Sodium tetra-borate anhydrous (borax) and dry toluene were supplied by Acros Organics. Fmoc-FFpY was purchased by PepMic, ammonium hydroxide by Carlo Erba and ethanol by VWR. Mueller Hinton (MH) broth was purchased from Merck (Germany).

### Synthesis and Functionalization of Silica Nanoparticles

The synthesis of silica nanoparticles (NP) and their functionalization with AP were carried out following the procedure described previously (Criado-Gonzalez et al., 2019a). After their synthesis, NPs were functionalized covalently by AP using an epoxy silane coupling agent, giving rise to NPs@AP (Supplementary Data). The catalytic activity of NPs@AP suspensions was determined employing PNP, a substrate which is transformed in *para*-nitrophenol (λ_*max*_ = 405 nm) by action of AP.

### Multilayer Film Preparation and Fmoc-FFpY Self-Assembly

All solutions were prepared in 25 mM borax buffer pH 9.5. Multilayer films were built on glass slides of 12 mm diameter (Marienfeld) or silicon slides of 10 × 10 mm (Sil’tronix Silicon Technologies). As the substrates are negatively charged, a precursor layer of branched PEI (1 mg.mL^–1^) was deposited to achieve a homogeneous distribution of positive charges over the surface allowing an efficient deposition of the coating. For that, substrates were immersed in a solution of PEI for 5 min followed by a rinsing step with borax buffer for 2 min. After that, the multilayer film was built up through sequential contact of the substrate with 400 μL of PSS (1 mg.mL^–1^) (polyanion) or PAH (1 mg.mL^–1^) (polycation) solutions for 5 min with a rinsing step in borax buffer of 2 min after each polyelectrolyte deposition. This cycle was repeated two times and samples were denoted as polyelectrolyte multilayer (PEM). Then the substrate was put in contact with 400 μL of NPs@AP (1.25% w/v in borax buffer) suspension for 60 min followed by three rinsing steps of 2 min in borax buffer. Samples were denoted as NPs@AP coatings. Subsequently, 400 μL Fmoc-FFpY (1 mg.mL^–1^) solution was brought in contact with NPs@AP coating for 16 h.

### Quartz Crystal Microbalance With Dissipation Monitoring (QCM-D)

Quartz crystal microbalance with dissipation monitoring experiments were performed in a QCM-D cell on a Q-Sense E1 apparatus (Q-Sense AB, Gothenburg, Sweden) at 22°C using an open cell. The resonance frequencies of a gold coated crystal and the dissipation factors at the fundamental frequency at 5 MHz (ν = 1) were monitored during the deposition of NPs@AP and the Fmoc-FFY self-assembly. The perturbation due to the pipetting of the solutions at each deposition steps were removed from the plot.

### Atomic Force Microscopy (AFM)

Atomic force microscopy Multimode Nanoscope IV (Bruker, Palaiseau, France) was used to analyze the surface topography of the coatings. Micrographs were recorded in Peak Force Tapping (ScanAsyst) mode by using silicon tips from Bruker (Model: ScanAsyst-Air) mounted on aluminum coated silicon nitride cantilevers. All samples were observed in dry state with triangular cantilevers having a spring constant of around 0.4 N.m^–1^ and a nominal tip radius of 2 nm. Selected AFM images were treated with the NanoScope Analysis Software (version 1.7). Samples were prepared on silicon substrates and air dried before analysis. Prior to the sample preparation, the silicon wafers were incubated in ethanol/water (50% by vol) mixture for 15 min and activated using plasma treatment for 3 min.

### Circular Dichroism (CD)

Circular dichroism (CD) spectra were recorded between 190 and 320 nm using a Jasco J-1100 spectropolarimeter with a data pitch of 1 nm on the light wavelength. Samples were built up on a quartz slide of 1 mm thickness.

### Fluorescence Spectroscopy

Fluorescence spectra were recorded between 300 and 355 nm using a Fluoromax-4 (Horiba Jobin Yvon – Edison, NJ, United States) at an excitation wavelength of 290 nm using a quartz slide of 1 mm thickness to build up the samples.

### Scanning Electron Microscopy (SEM) and Cryo-SEM

The Fmoc-FFY hydrogel coating on a Silicon wafer was placed on a home-made cryo-holder (Vigier-Carrière et al., 2017) to be quickly plunged into an ethane slush. As the sample is free standing over the holder, the sample is rapidly frozen during the plunging by direct contact with the liquid ethane. Subsequently, the sample is transferred into the Quorum PT 3010 chamber attached to the microscope. There, the frozen sample is fractured with a razor blade. A slight etching at −90°C may be performed to render the fibers more visible. The sample is eventually transferred in the FEG-cryo SEM (Hitachi SU8010) and observed at 1 kV at −150°C.

### Inverted Tube Tests

All solutions were prepared in 25 mM borax buffer pH = 9.5. 150 μL of Fmoc-FFpY (1 mg.mL^–1^) was mixed with 50 μL of NPs@AP (5% w/v) in vials. After 24 h, inverted tube tests were carried out to determine the hydrogel formation. Then, 400 μL of bacteria culture medium (MH) was added and the stability was checked up to 9 days.

### Antibacterial Activity

Antibacterial tests were carried out employing one strain Gram-positive bacteria, *Staphylococcus aureus* (*S. aureus*, ATCC 25923) and another strain Gram-negative bacteria, *Escherichia coli* (*E. coli*, ATCC 25922). *S. aureus* and *E. coli* were precultured separately in aerobic conditions at 37°C in a MH broth medium (Merck, Germany), pH 7.4. One colony from previously prepared agar plates by bacteria streaking protocol was transferred to 7 mL of MH medium and incubated in an agitator overnight at 37°C. To obtain bacteria in their mid-logarithmic phase of growth, the absorbance at 620 nm (OD_620_) of overnight cultures was adjusted to 0.001 by diluting in MH, corresponding to a final cell density of approximately 8 × 10^5^ CFU.mL^–1^. Cultures growing in the presence of antibiotics (Tetracycline and Cefotaxime) were taken as positive control. Bacteria quantification (in colony forming unit per mL, CFU.mL^–1^) was performed at time zero and 24 h after the incubation with the samples. This was determined by plating 100 μL of the supernatant, after serial dilution, on nutrient agar plates at 37°C overnight, then the viable cell colonies were counted and represented as log_10_ reduction (CFU.mL^–1^). To determine the Minimum Inhibitory Concentration (MIC) value of Fmoc-FFpY and Fmoc-FFY hydrogel, the peptide was dissolved in RPMI medium at 2 mg.mL^–1^ and at lower concentrations by serial dilution in RPMI. *S. aureus* was pre-cultured overnight in MH medium, and diluted in RPMI medium. MIC value of Fmoc-FFpY was determined by mixing 100 μL of the peptide solution and 100 μL of *S. aureus* suspension with a final optical density of OD = 0.001. To form the Fmoc-FFY hydrogel, 100 μL of the peptide solution (at different concentrations) was mixed with 100 μL of AP solution (1.4 μg.mL^–1^) and left 24 h at 22°C. MIC value of Fmoc-FFY hydrogel was determined by adding to the hydrogel 200 μL of *S. aureus* inoculation with a final optical density of OD_620_ = 0.001. Fmoc-FFY hydrogel coating samples were built up on glass substrates of 12 mm diameter, placed in 24-well plates and sterilized for 20 min by UV light. 400 μL of *S. aureus* or *E. coli* inoculation (OD_620_ = 0.001) were added to each well and incubated at 37°C for 24 h. In repetitive culture experiments, the supernatant was totally removed and replaced by 400 μL of freshly prepared *S. aureus* or *E. coli* inoculation (OD_620_ = 0.001). Regarding the statistical analysis, all experiments were carried out independently in triplicate and three analyses per replication at least were done. The significant differences in the experimental data were analyzed using the ANOVA procedure (SAS Institute Inc., Cary, NC, United States) at *p* < 0.01 with mean separation determined by Tukey’s multiple range tests.

### Cell Cytotoxicity Test

Cytotoxicity assays were carried out by incubating the gels with 1 mL of DMEM at 37°C. After 24 and 48 h, the extracts were removed under sterile conditions. Separately, NIH 3T3 mouse embryonic fibroblasts cells were seeded at a density of 1 × 10^5^ cells.mL^–1^ in complete medium in a sterile 24 well culture plate and incubated to confluence. After 24 and 48 h of incubation, the medium was replaced with the corresponding extracts and incubated at 37°C in humidified air with 5% CO_2_ for 24 h and 48 h. Subsequently, plates were incubated with 500 μL per well of a MTT solution (0.1% w/v 3-(4,5-dimethylthiozol-2-yl)-2,5-diphenyltetrazolium bromide in Phosphate Buffer Saline) and incubated for 180 min at 37°C. Medium was displaced by 500 μL of DMSO. Optical Density (OD) was measured at 570 nm. The cell viability was calculated from Eq. (1):

(1) $$Cellviability\left(\%\right)=\frac{O\,D_{S}-O\,D_{B}}{O\,D_{C}-O\,D_{B}}\cdot 100$$

where OD_*S*_, OD_*B*_, and OD_*C*_ are the optical density for the sample (S), blank (culture medium) (B), and control (glass) (C), respectively.

## Results and Discussion

### Physico-Chemical Characterization of Fmoc-FFY Hydrogel Coating

**FIGURE 1:**
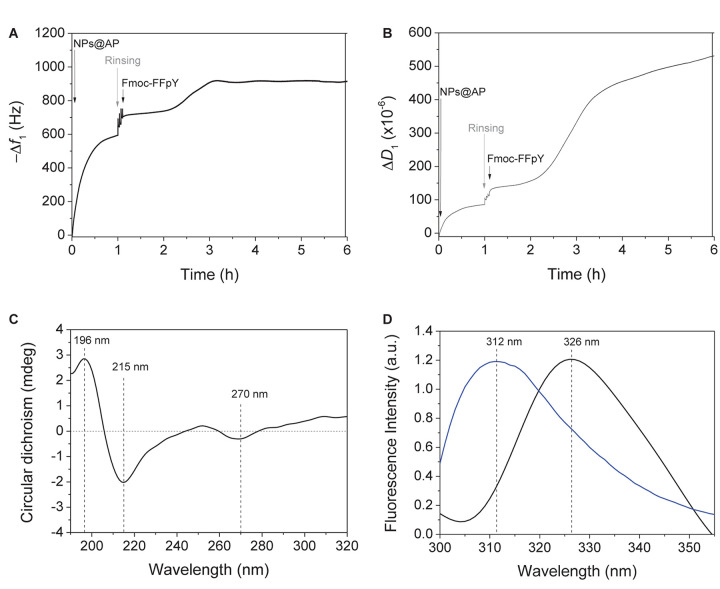
Evolution of the **(A)** fundamental frequency shift and **(B)** dissipation value, measured by QCM-D at 5 MHz, during the deposition of NPs@AP on PEM precursor film, leading to NPs@AP coating, followed by the contact with Fmoc-FFpY solution leading to the Fmoc-FFY hydrogel coating. **(C)** Circular Dichroism and **(D)** Fluorescence emission (λ_*ex*_ = 290 nm) spectra of Fmoc-FFY hydrogel coating (black curve) and Fmoc-FFpY solution (blue curve).

Alkaline phosphatase functionalized silica nanoparticles suspensions were obtained with a solid content of 5% (w/v) and had an average diameter of 132 ± 10 nm (Supplementary Figure S1). The suspension showed an activity equivalent to 30 units.mL^–1^ determined using PNP, a model substrate of AP, with a calibration curve (Supplementary Figure S2). To immobilize NPs@AP on a planar surface, a PEI-(PSS/PAH)_2_ polyelectrolyte multilayer film (PEM) was first deposited as precursor film. When NPs@AP suspension is put in contact with the PEM precursor film, a high increase of the normalized frequency shift is observed by QCM-D reaching about 700 Hz after the rinsing steps (Figure 1A).

The obtained NPs@AP coating has a catalytic activity equivalent to 0.6 μg.mL^–1^ of AP in solution, corresponding to 0.08 μg.cm^−2^ of active enzyme. AFM images of NPs@AP coating showed round shape NPs all over the surface (Supplementary Figure S3). The section profile of the AFM images provides an average diameter of the deposited NPs of 140 nm (Supplementary Figure S3C) in agreement with SEM observations (Supplementary Figure S1). The NPs@AP coating was then employed as catalytic support for the localized growth of supramolecular peptide by putting it in contact with Fmoc-FFpY solution (1 mg.mL^–1^) (Scheme 1). The Fmoc-FFY self-assembly induced an increase of the frequency shift of about 230 Hz after 3.5 h (Figure 1A). The dissipation value reached 400 × 10^–6^ which is the signature of highly hydrated films (Figure 1B). In contrast, NPs@AP coating had a dissipation value of 130 × 10^–6^. By decreasing Fmoc-FFpY concentration of the solution in contact with NPs@AP coating, the final frequency shift decreased reaching 100 Hz at 0.5 and 0.25 mg.mL^–1^ and 50 Hz at 0.1 mg.mL^–1^. This was corroborated by the decrease of the dissipation value (Supplementary Figures S4A,B). As a control experiment, the contact of a non-self-assembling peptide, Fmoc-G-OH (1 mg.mL^–1^), with the NPs@AP coating induces an increase of 43 Hz of the frequency shift (Supplementary Figures S4C,D). Fmoc-G-OH peptide is probably physisorbed on the surface such as Fmoc-FFpY when the concentration is lower than 0.1 mg.mL^–1^.

The structural organization of Fmoc-FFY self-assembled on NPs@AP coating, named Fmoc-FFY hydrogel coating, was investigated by CD (Figure 1C and Supplementary Figure S5). The CD spectrum shows a positive band at 196 nm and a strong negative band at 215 nm characteristics of β-sheets structures (Smith et al., 2008). The presence of a negative band at 270 nm is assigned to offset face-to-face stacking of the Fmoc moieties which is not observed in the case of non-self-assembling Fmoc-FFpY in solution (Ryan et al., 2010). The excimer formation of Fmoc moieties was checked after Fmoc-FFY self-assembly by fluorescence spectroscopy (Tang et al., 2011; Criado-Gonzalez et al., 2019b). When excited at 290 nm, Fmoc-FFpY solution have a fluorescence emission peak at 312 nm due to fluorenyl moieties (Figure 1D). The self-assembly of Fmoc-FFY on NPs@AP coating induced a shift of this peak up to 326 nm, due to fluorenyl excimers.

### Morphological Characterization of Fmoc-FFY Deposited Hydrogel

**FIGURE 2:**
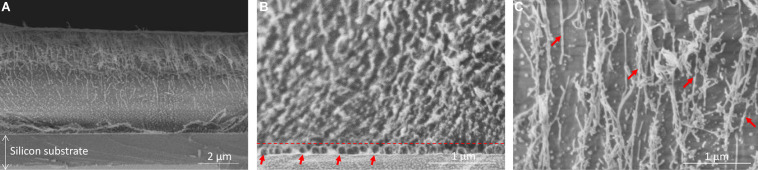
Cryo-SEM images of **(A)** Fmoc-FFY hydrogel self-assembled on NPs@AP coating, **(B)** zoom-in of the bottom area to show the localization of the NPs@AP delimited with a dashed line and highlighted with red arrows and **(C)** zoom-in of the upper part showing Fmoc-FFY fibers (red arrows).

The morphology of the Fmoc-FFY hydrogel coating was visualized by Cryo-SEM (Figure 2). The cross-section of the coating showed three different areas. The magnification of the bottom part allowed to distinguish monolayer and multilayers of round shape NPs@AP, with a size of ∼ 120 nm determined using ImageJ, on the surface of the glass slide (Figure 2B). It can be noticed that there is no diffusion of NPs@AP throughout the whole Fmoc-FFY hydrogel after 16 h in contact (dashed line in Figure 2B). Fmoc-FFY fibers grow from the NPs@AP giving rise to a hydrogel coating with a thickness of ∼ 5 μm. A gradient of organic matter is observed in the self-assembled Fmoc-FFY hydrogel with two different areas (Figure 2A). Higher density of fibers is close to the bottom, in direct contact NPs@AP, and a lower density on the upper part of the Fmoc-FFY hydrogel. A magnification of the upper part allows to distinguish long fibers of micrometer length oriented in the vertical direction perpendicular to the substrate (Figure 2C). Fmoc-FFY fibers have a diameter ranging from 13 to 40 nm (red arrows in Figure 2C).

### Antimicrobial Properties of Fmoc-FFY Hydrogel Coating

**FIGURE 3:**
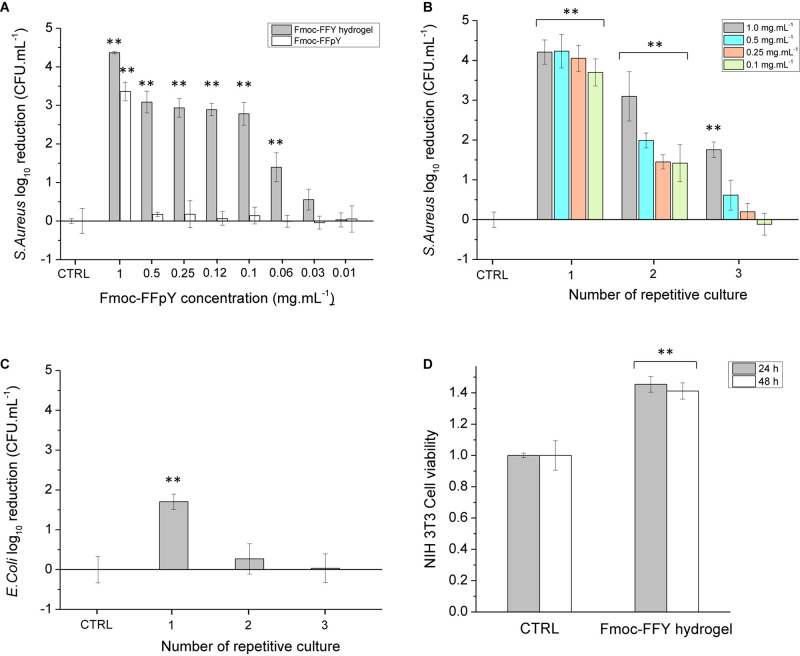
Colony-forming unit (CFU) log_10_ reduction on planktonic **(A)** *S. aureus* cells incubated in the presence of different concentrations of Fmoc-FFpY and of Fmoc-FFY hydrogel, self-assembled with different concentrations of Fmoc-FFpY, **(B)** *S. aureus* cells incubated on Fmoc-FFY hydrogel coatings, self-assembled with different concentrations of Fmoc-FFpY on NPs@AP coating and **(C)** *E. coli* cells incubated on Fmoc-FFY hydrogel coating, self-assembled with 1 mg.mL^– 1^ Fmoc-FFpY solution on NPs@AP coating, as a function of the number of repetitive culture. The coating was brought in contact with a fresh pathogen suspension for 24 h. In repetitive culture experiments, the supernatant was removed and replaced every 24 h by a fresh suspension. **(D)** Cytotoxicity assay of Fmoc-FFY gel through MTT indirect test using NIH 3T3 mouse embryonic fibroblasts cells. Uncoated glass substrates were employed as controls (CTRL). Diagrams include the results from three independent experiments in triplicate and results are shown as mean ± s.e.m. and the ANOVA results at a significance level of **p < 0.01 vs. uncoated glass (negative control, CTRL).

Before to study the antibacterial properties of the Fmoc-FFY hydrogel coating, the stability of the hydrogel was tested in contact with bacteria culture medium. For that purpose, Fmoc-FFY hydrogel was formed in a vial by mixing Fmoc-FFpY solution and NPs@AP suspension. The inverted tube test indicated the formation of a gel after 24 h (Supplementary Figure S6). The hydrogel remains stable up to 9 days in contact with MH culture medium. To underline the necessity of the enzymatic dephosphorylation of the peptide, the antibacterial properties of Fmoc-FFpY and Fmoc-FFY hydrogel were studied against Gram-positive *S. aureus* in RPMI media at different concentrations in peptide (Figure 3A). Bacteria quantification (in colony forming unit per mL, CFU.mL^–1^) was performed at time zero and 24 h after the incubation with the samples and presented in log_10_ reduction. At 1 mg.mL^–1^, 3-log_10_ (99%) reduction bacterial load against *S. aureus* is obtained for Fmoc-FFpY peptide, whereas 4-log_10_ (99.99%) reduction were achieved with Fmoc-FFY hydrogel, obtained by dephosphorylation of the peptide. At lower peptide concentrations (≤0.5 mg.mL^–1^), the antibacterial property of Fmoc-FFpY is lost whereas Fmoc-FFY hydrogel showed 3-log_10_ (99%) reduction of *S. aureus* suspension up to 0.1 mg.mL^–1^ of peptide. The self-assembly of the peptide ensure the antibacterial effect at low concentration. Bare NPs@AP coatings did not prevent the proliferation of the bacteria (Supplementary Figure S7). The antibacterial effect of the Fmoc-FFY hydrogel coating, self-assembled on NPs@AP at different concentrations in Fmoc-FFpY, was tested against *S. aureus* (Figure 3B). After 24 h of contact, Fmoc-FFY hydrogel coatings demonstrated efficacy of more than 4-log_10_ (99.99%) reduction of bacterial load against *S. aureus* whatever the concentration of peptide used for their self-assembly up to 0.1 mg.mL^–1^. To mimic the worst conditions of repetitive bacterial infections, for example in the case of urinary or venous catheter-associated infections, the bacterial inoculation in contact with the hydrogel coating was completely renewed every 24 h. At least 3-log_10_ (99%) reduction of *S. aureus* was observed for the second culture in contact with Fmoc-FFY hydrogel coating self-assembled with 1 mg.mL^–1^ Fmoc-FFpY. At lower concentration in peptide, the second culture with *S. aureus* showed between 2 and 1-log_10_ (90%) reduction. In the following culture, a significant decrease in efficiency is detectable with 1-log reduction only with 1 mg.mL^–1^. Fmoc-FFY hydrogel coating, self-assembled with 1 mg.mL^–1^ Fmoc-FFpY, showed only 1-log reduction of bacterial load against Gram-negative *E. coli* after 24 h with no significant inhibition at the second culture (Figure 3C). A selective antibacterial effect of Fmoc-FFY hydrogel coating was observed against *S. aureus* Gram-positive bacteria in contrast to *E. coli* Gram-negative bacteria.

The mechanism of action of Fmoc-FFY hydrogels could be attributed to a combination of two factors: the fibrillary β-sheet structure of the self-assembly and the peptide hydrophobicity as it was reported for β-amyloid peptides (Kagan et al., 2012; Last and Miranker, 2013). Pioneering works of Moir and coll. demonstrated the antimicrobial activity of Alzheimer’s β-amyloid peptides *in vitro* and *in vivo* (Soscia et al., 2010; Kumar et al., 2016). Diphenylalanine moieties (FF), identified as the core recognition motif of β-amyloid peptides, were reported to self-assemble in discrete and stiff nanotubes adopting β-sheet structures (Reches and Gazit, 2003). Self-assembled FF peptides have the ability to bind to the surface of bacterial membrane, to aggregate and to orient themselves to optimize hydrophilic/hydrophobic interactions, inducing a surface tension leading to membrane depolarization, pore formation and release of cellular content (Schnaider et al., 2017). Moreover, FF peptides also induce upregulation of stress response regulons at sub-lethal concentration causing severe damage to bacterial morphology. Recently, the antibacterial activity of self-assembled Fmoc-F was attributed to the peptide release from the hydrogel inducing oxidative and osmotic stress as well as altering bacterial membrane integrity (Gahane et al., 2018). The comparison of Fmoc-FF cationic peptide, designed with a pyridinium moiety at the C-terminal, with other Fmoc-cationic peptides showed that the proximity of fluorenyl and phenyl moieties achieves an optimum hydrophobicity environment improving the antibacterial activity (Debnath et al., 2010). In summary, the antibacterial property of the Fmoc-FFY hydrogel coating could be explained by the ability of the β-sheet self-assembled nanofibers to bind and aggregate on the surface of the bacterial membrane, altering the membrane integrity and also to oxidative and osmotic stresses leading to the death of the bacteria. This mechanism is confirmed by the efficiency of peptide self-assembly at low concentration in comparison to the non-self-assembled peptide. In contrast to Gram-positive, Gram-negative bacteria have an additional outer bilayer membrane, formed by lipopolysaccharides and phospholipids, that makes difficult the diffusion of molecules across the membrane and renders them more resistant to break (Haldar et al., 2005). The cytotoxicity of the Fmoc-FFY gel was determined *in vitro*, through a MTT indirect test, employing NIH 3T3 mouse embryonic fibroblasts cells (Figure 3D). NIH 3T3 cell viability do not show any decrease of up to 48 h proving that Fmoc-FFY gel is not cytotoxic. This result confirmed the absence of toxicity of Fmoc-FF peptides when they are in self-assembled form (Truong et al., 2015). Besides, Fmoc-FF peptides have been used as 3-D cell culture scaffolds for chondrocytes (Jayawarna et al., 2006) or astrocytes (Liebmann et al., 2007).

## Conclusion

In this work a phosphorylated peptide, Fmoc-FFpY, able to be solubilized in water at room temperature, was transformed in Fmoc-FFY hydrogelator by action of NPs@AP immobilized on a surface. The hydrogel coating, based on fibers homogeneously distributed all around the sample, was efficient to prevent *S. aureus* growth with 4-log_10_ of viable bacteria. This coating could serve in infections were Gram positive bacteria are prevalent, e.g., intravascular catheter infections. This kind of antibacterial self-assembled hydrogel represents a very simple and efficient method to coat biomaterial surfaces for protection against bacteria proliferation. In clinical practice to ensure the integrity of the hydrogel coating, one idea would be to trigger the peptide self-assembly *in situ* after implantation of the medical device by addition of the peptide solution near the enzyme functionalized implant surface. With the prospect of long-term use of the antibacterial coating, the surface triggered self-assembly of peptide could be done on porous surfaces or even inside the pores of foams. The high surface of coverage, obtained on rough or porous implants, could lead to sustained antibacterial property as the coating maintained its property even after being in contact several times with fresh inoculated *S. aureus* suspension. Moreover in a curative strategy, the peptide self-assembly could be triggered by using enzymes naturally secreted by bacteria to obtain the antibacterial gel either inside the cells or at their surface using overexpression of phosphatase by bacteria (Yang et al., 2007) or by mammalian cancer cells (Pires et al., 2015).

## Data Availability Statement

All datasets presented in this study are included in the article/Supplementary Material.

## Author Contributions

PS and FB conceived the project. MC-G, LJ, PS, and FB designed the experiments. MC-G carried out the preparation of all samples under study, QCM-D analyses, gelation tests, circular dichroism, and fluorescence spectroscopy characterization. MI performed the AFM characterization and bacteria tests. MC-G, MI, and FB contributed to the interpretation of the results. AC and MS carried out the SEM and cryo-SEM experiments. All authors contributed to the article and approved the submitted version.

## Conflict of Interest

The authors declare that the research was conducted in the absence of any commercial or financial relationships that could be construed as a potential conflict of interest.
