# Supplementary material for: Surface Triggered Self-Assembly of Fmoc-Tripeptide as an Antibacterial Coating
Source: Front Bioeng Biotechnol. 2020 Aug 7;8:938. doi: 10.3389/fbioe.2020.00938 (PMC7438842; doi:10.3389/fbioe.2020.00938)
Supplement: Supplementary file 1 [file Data_Sheet_1.docx]

Supplementary Material

Surface Triggered Self-assembly of Fmoc-tripeptide as an Antibacterial Coating

Miryam Criado-Gonzalez^1,2*^, Muhammad Haseeb Iqbal^1, 2^, Alain Carvalho^1^, Marc Schmutz^1^, Loïc Jierry^1^, Pierre Schaaf^1,2,3^, Fouzia Boulmedais^1*^

^1^Université de Strasbourg, CNRS, Institut Charles Sadron UPR 22, 67034 Strasbourg, France.

^2^Institut National de la Santé et de la Recherche Médicale, UMR-S 1121, “Biomatériaux et Bioingénierie”, 67087 Strasbourg, France

^3^Université de Strasbourg, Faculté de Chirurgie Dentaire, Fédération de Médecine Translationnelle de Strasbourg and Fédération des Matériaux et Nanoscience d’Alsace, 67000 Strasbourg, France

*** Correspondence:** Corresponding Authors: [fouzia.boulmedais@ics-cnrs.unistra.fr](mailto:fouzia.boulmedais@ics-cnrs.unistra.fr); miryamcg22@gmail.com

# Supplementary Data

**Synthesis and functionalization of silica nanoparticles**

Silica nanoparticles (NPs) were synthetized following the procedure described previously (Criado-Gonzalez, 2019, *Langmuir* 35**,** 10838-10845). Briefly, 80 mL of ethanol, 4.85 mL of Milli-Q water and 3.6 mL of NH4OH were mixed in a two necks flask under mechanical stirring (400 rpm) and heated up to 55°C. When the temperature is reached, 8 mL of ethanol and 3.1 mL of TEOS, mixed in a separated vial, were added quickly to the previous solution and kept under stirring for 5 h. In a second step in order to obtain larger nanoparticles from the first synthesis (~ 80 nm), 10 mL of the NPs colloidal suspension were mixed with 70 mL ethanol, 13 mL Milli-Q water and 7.5 mL of NH4OH in a two necks flask and heated again up to 55°C under mechanical stirring (400 rpm). When the temperature is reached, a mixture of 10 mL of ethanol and 1 mL of TEOS was added dropwise to the solution and kept under stirring for 5 h. Subsequently, the solution was centrifuged (6000 rpm, 20 min) and the precipitate of NPs was washed three times with ethanol by centrifugation (6000 rpm, 20 min). For the covalent immobilization of AP, 2.5 mL of NPs were dried at 100°C under argon atmosphere for 2 h in a 50 mL flask. Then, 10 mL of 5%v/v GPMS in dry toluene were added to dry NPs. The suspension was sonicated for 10 min to avoid aggregates and kept shaking at 720 rpm overnight at room temperature. The obtained epoxy-functionalized NPs were centrifuged and the precipitate was firstly washed three times with toluene to remove unbounded GPMS and subsequently, three times with ethanol to remove toluene. The final NPs were dispersed in 1 mL of ethanol and subsequently added dropwise to an aqueous solution of AP (1 mg.mL-1 in borax buffer (25 mM, pH = 9.5)) and kept shaking for 24h at 4°C. Then, NPs were centrifuged and washed five times with borax buffer (25 mM, pH = 9.5) to remove the unbounded enzyme. NPs@AP showed an activity equivalent to 30 units.mL^-1^ with a solid content of 5% w/v. Finally, NPs@AP were redispersed in 1 mL borax buffer (25 mM, pH = 9.5) and stored at 4°C until needed.

# Supplementary Figures

**Supplementary Figure 1.** (A) SEM micrograph of NPs@AP and (B) histogram obtained by counting the 46 nanoparticles using ImageJ.

**Supplementary Figure 2.** Catalytic activity tests (A) Evolution of the absorbance, measured at 405 nm, as a function of time of NPs@AP suspension in contact with PNP solution (B) Calibration curve obtained by measuring the slope of the evolution of the absorbance, measured at 405 nm, as function of time of AP in solution in contact with PNP. (C) Evolution of the absorbance, measured at 405 nm, as a function of time of NPs@AP coating. PNP is transformed by AP into *para*-nitrophenol (λ_max_ = 405 nm). The dashed line indicates the tangent of the curve where slope was determined to calculate the AP activity.

**Supplementary Figure 3.** AFM images of (A) NPs@AP coating and (B) zoom-in to distinguish NPs@AP with (C) the section profile of the area marked with a red line in figure B.

**Supplementary Figure 4.** Evolution of the (A) normalized frequency shift and (B) dissipation value as a function of time when NPs@AP coating is put in contact with different concentrations of Fmoc-FFpY. Evolution of the (C) normalized frequency shift and (D) dissipation value as a function of time when NPs@AP coating is put in contact with Fmoc-FFpY (black curve) and Fmoc-G-OH (red curve) solutions.

**Supplementary Figure 5.** HT voltage curves of Fmoc-FFY hydrogel coating.

**Supplementary Figure 6.** Inverted tube tests of hydrogels formed by mixing NPs@AP (1.25% w/v) with Fmoc-FFpY (1.0 mg.mL^-1^) before and after contact with 400 µL of bacteria culture medium (MH). No evolution of the gel appears in the presence of culture medium as it marked with the dashed red line.

**Supplementary Figure 7.** Normalized *S. Aureus* and *E. Coli* growth after 24 h contact with NPs@AP coating. The normalization was performed with respect to OD_620_ measured on a bare glass substrate taken as 100% growth.
